# Human In Vivo Cardiac Magnetic Resonance Imaging at 7 T: Feasibility, Applications, and Current Limitations—A Systematic Review

**DOI:** 10.3390/diagnostics16060937

**Published:** 2026-03-22

**Authors:** Arosh S. Perera Molligoda Arachchige, Gabriel Amorim Moreira Alves, Ayça Zal, Giulia D’Acunto, Maciej Węglarz, Oana-Georgiana Voicu, Erica Maffei, Filippo Cademartiri

**Affiliations:** 1 Università degli Studi di Milano, Via Festa del Perdono 7, 20122 Milan, Italy; 2 Faculty of Medicine & Surgery, Humanitas University, 20089 Rozzano, Italy; 3 Department of Medical Biotechnology and Translational Medicine, International Medical School, Università degli Studi di Milano, 20054 Milan, Italy; 4 Dipartimento di Scienze della Salute, Università degli Studi di Milano, 20054 Milan, Italy; 5 IRCCS SYNLAB SDN, 80143 Naples, Italy

**Keywords:** 7-Tesla, MRI, myocardial, applications, human, cardiovascular, heart

## Abstract

**Background/Objectives:** Cardiac magnetic resonance (CMR) imaging at 7 Tesla provides a substantially higher intrinsic signal-to-noise ratio compared with conventional 1.5 T and 3 T systems, potentially enabling higher spatial resolution, improved tissue contrast, and advanced metabolic imaging. However, clinical translation remains limited by technical challenges associated with ultra-high-field operation. This systematic review aimed to synthesize current human in vivo evidence on the feasibility, applications, and methodological limitations of 7-T cardiovascular MRI. **Methods:** A PRISMA-guided systematic search of PubMed, Cochrane Library, Web of Science, and Scopus was conducted from database inception through January 2025. Studies reporting human in vivo cardiovascular MRI at 7 Tesla were included. Data regarding study design, sample characteristics, imaging applications, feasibility, quantitative findings, and reported limitations were extracted and qualitatively synthesized. **Results:** Sixty-five studies met inclusion criteria, predominantly small prospective cohorts (mean sample size = 13), largely involving healthy volunteers. Across diverse applications—including coronary MR angiography, cine imaging, valvular assessment, vascular imaging, flow quantification, myocardial tissue characterization, and multinuclear (^31^P, ^23^Na, ^39^K) imaging—7-T CMR was consistently feasible and capable of producing high-quality images. Quantitative ventricular and vascular measurements were generally concordant with lower field strengths. Incremental benefits were most apparent in high-resolution structural imaging and metabolic applications, whereas routine functional and flow assessments showed limited additional advantages. No serious adverse events were reported. **Conclusions:** Human cardiovascular MRI at 7 Tesla represents a technically feasible research and early translational platform with selective advantages over established field strengths. Further advances in radiofrequency technology, protocol harmonization, and larger disease-focused studies are required to clarify its potential clinical role.

## 1. Introduction

The introduction of 7 T MRI into human cardiovascular imaging represents one of the most significant technological shifts in the evolution of cardiac/cardiovascular magnetic resonance (CMR) over the past two decades. At its core, the motivation for employing 7 T in CMR is driven by the substantial increase in intrinsic signal-to-noise ratio. This benefit can be directly converted into finer spatial resolution, improved temporal resolution, or a variable combination of both. The ability to obtain highly detailed visualization of the myocardium, coronary arteries, and valvular structures is an appealing prospect, especially considering the persistent technical barriers encountered at 1.5 T and 3 T [1]. Coronary artery imaging has historically struggled with low vessel contrast and complex motion patterns. Similarly, the resolution of conventional cine and tissue characterization techniques remains insufficient for detecting subtle microvascular alterations or small-scale fibrosis [2]. Ultra-high-field imaging provides an opportunity to overcome these limitations.

Following the clinical maturation of 3 T cardiovascular MRI, intermediate ultra-high-field systems operating at 5 T have emerged as a potential bridge between conventional and ultra-high-field imaging. Early human studies suggest that 5 T CMR is feasible and provides a higher signal-to-noise ratio compared with 3 T, enabling improved spatial resolution while maintaining reliable ventricular quantification. However, current evidence indicates that 5 T does not introduce fundamentally new cardiovascular MRI capabilities beyond those achievable at lower field strengths. In contrast, 7 T offers substantially greater signal gains and enhanced spectral separation, supporting ultra-high-resolution structural imaging and multinuclear applications that remain impractical at 5 T [3,4,5].

However, the transition to 7 T introduces characteristic challenges that are not encountered at lower fields. Radiofrequency energy distribution becomes markedly non-uniform due to wavelength shortening within biological tissues, producing areas of signal dropout and unpredictable contrast behavior. These inhomogeneities also contribute to higher energy deposition, reflected in stricter SAR limits that restrict sequence design. Standard inversion pulses used in T1 mapping become less effective, and many clinically established sequences require considerable adaptation or replacement [6,7,8]. Because of these constraints, early research at 7 T focused predominantly on engineering developments, including coil design, B1-shimming strategies, and parallel transmit technologies. Only in recent years has the field progressed into systematic cardiac imaging studies.

Given this transition from technical demonstration to early clinical feasibility, a comprehensive synthesis of human 7 T CMR studies has become necessary. While individual investigations have highlighted potential benefits in coronary angiography, cine imaging, myocardial mapping, valve assessment, and vascular flow quantification, the broader landscape remains fragmented. Furthermore, the diversity of imaging platforms, coil architectures, and transmission schemes complicates the interpretation and comparison of results across studies [9]. A focused systematic review serves not only to consolidate the evidence but also to delineate the specific niche in which 7 T CMR might eventually provide added value beyond 3 T imaging.

This review therefore seeks to evaluate the current state of 7 T CMR by examining human in vivo applications, technical developments, and emerging clinical insights. It aims to describe common themes across studies, highlight observed limitations, and provide perspective on future directions that may facilitate the transition from feasibility research to routine clinical usage. The findings from this review will assist clinicians, imaging scientists, and industry partners in understanding the practical potential and remaining challenges of 7 T cardiovascular imaging.

## 2. Methods

### 2.1. Research Question (PIRD Framework)

This systematic review was structured according to a diagnostic PIRD framework.

**Population (P):** Human participants undergoing cardiovascular magnetic resonance imaging.
**Index Test (I):** 7-Tesla cardiovascular MRI across any application (e.g., cine imaging, coronary MRA, myocardial tissue characterization, perfusion, flow imaging, spectroscopy).
**Reference Standard (R):** When available, comparator imaging included 1.5 T or 3 T MRI; several feasibility studies reported no comparator.
**Domain (D):** Feasibility, image quality, diagnostic accuracy, quantitative performance, technical implementation, and safety of 7 T cardiovascular MRI.

A traditional PICO framework was not appropriate because most included studies were early-phase feasibility investigations with heterogeneous outcomes and variable or absent comparators. The PIRD framework is therefore used to define the review question and ensure alignment with diagnostic imaging methodology standards.

### 2.2. Protocol and Registration

A review protocol was developed a priori to define the research question, eligibility criteria, search strategy, and planned methods for study selection and data extraction. The protocol was not prospectively registered in PROSPERO, as the review focuses on early-phase feasibility and technical cardiovascular magnetic resonance investigations, which do not involve standardized clinical outcomes or quantitative meta-analysis typically required for PROSPERO registration categories. To ensure transparency, the full protocol is provided as Supplementary Material (Supplementary File S2). All methodological steps—including study eligibility assessment, literature search, screening, and data extraction—were conducted in accordance with the predefined protocol. No major methodological deviations occurred. Minor clarifications to the methodological appraisal framework were made to improve transparency and reporting consistency; these refinements did not alter eligibility criteria, search strategy, or study selection.

### 2.3. Search Strategy

Our search strategy adhered to PRISMA guidelines and was designed to capture all published human cardiovascular MRI studies performed at 7 T [10]. Searches were executed in PubMed, Cochrane Library, Web of Science and Scopus from inception to January 2025. The following search string was used for PubMed: ((“magnetic resonance imaging”[MeSH Terms] OR MRI[tiab] OR “MR imaging”[tiab]) AND (“7 Tesla”[tiab] OR “7 T”[tiab] OR “ultra high field”[tiab] OR “ultrahigh field”[tiab]) AND (cardiac[tiab] OR heart[tiab] OR myocard*[tiab] OR coronary[tiab] OR aortic[tiab] OR cardiovascular[tiab] OR “cardiac magnetic resonance”[tiab] OR CMR[tiab])), and was subsequently adapted to the format required for the remaining databases. Only articles published in the English language were included. We did not identify additional articles through citation tracking and reference list review.

### 2.4. Eligibility Criteria

Studies were eligible if they involved human participants undergoing 7 T cardiovascular MRI and reported at least one of the following outcomes: anatomical imaging, functional assessment, hemodynamic measurement, quantitative tissue characterization, image quality evaluation, or clinical feasibility. Engineering papers that presented only coil design or electromagnetic simulations without human imaging results were excluded. Studies performed exclusively in animals, ex vivo, post-mortem or in phantoms were also excluded.

Studies were included if they investigated clinical applications of 7-T MRI of the heart or central and systemic cardiovascular system, including the myocardium, great vessels, pulmonary arteries, carotid arteries, and systemic peripheral arteries of the upper and lower extremities (e.g., hand or foot vasculature).

Technical feasibility studies focusing on RF transmission strategies or coil configurations were included when in vivo human cardiac imaging was performed and were categorized according to their primary imaging application.

Studies focusing exclusively on intracranial neurovascular imaging (e.g., stroke, basilar artery) or organ-specific non-cardiac vasculature (e.g., renal or mesenteric arteries) were excluded.

### 2.5. Study Selection and Data Extraction

Two reviewers independently screened all titles and abstracts using Zotero (6.0.26), followed by a full-text review of potentially eligible articles [11]. Disagreements at any stage (screening or extraction) were resolved through structured discussion; if consensus could not be reached, a senior author adjudicated the final decision. Data extraction from each potentially eligible study was also performed independently by two reviewers using a Microsoft Excel Workbook for subsequent analysis. This extraction included publication year, country, study population, sample size, imaging application, comparator field strengths, described image quality or feasibility outcomes, artifacts or limitations, and technical setup including scanner manufacturer, coil design and use of parallel transmit technology [12].

### 2.6. Risk of Bias and Methodological Quality

Given the predominance of early-phase feasibility investigations, a structured qualitative methodological appraisal framework was applied rather than QUADAS-2, which is primarily designed for diagnostic accuracy studies with clearly defined index tests, reference standards, and clinical outcomes, and may not be fully applicable to exploratory technical imaging investigations without standardized comparators or accuracy endpoints [13]. Methodological literature describing the phased evaluation of diagnostic tests emphasizes that early-stage technical development studies differ fundamentally from later-stage diagnostic accuracy and outcome-validation studies, and may require alternative appraisal strategies focused on technical maturity, reproducibility, and transparency rather than bias scoring alone [14,15,16]. Accordingly, studies were categorized based on study maturity rather than formal bias domains. Quantitative validation studies required comparator field strength, reproducibility metrics, adequate sample size (≥10), and transparent acquisition reporting. Comparative feasibility studies included a field-strength comparator but lacked full validation metrics. Exploratory feasibility studies were early-phase investigations without comparator validation or reproducibility assessment. When primary studies incompletely reported methodological details, this was recorded as “not reported” and reflected in the structured methodological appraisal. Results are summarized in Supplementary File S1 (“methodological quality appraisal” sheet).

### 2.7. Data Synthesis

Owing to substantial heterogeneity across study designs, imaging protocols and outcomes, no meta-analysis was performed. Results were synthesized narratively and thematically to characterize the evolving applications of 7-Tesla cardiovascular MRI. Feasibility was operationally defined as successful acquisition of diagnostic-quality images and completion of the intended imaging protocol. Image quality outcomes were recorded as reported by the original investigators, including quantitative metrics (e.g., SNR, CNR, vessel sharpness) or structured qualitative scoring when available.

## 3. Results

**Figure 1:**
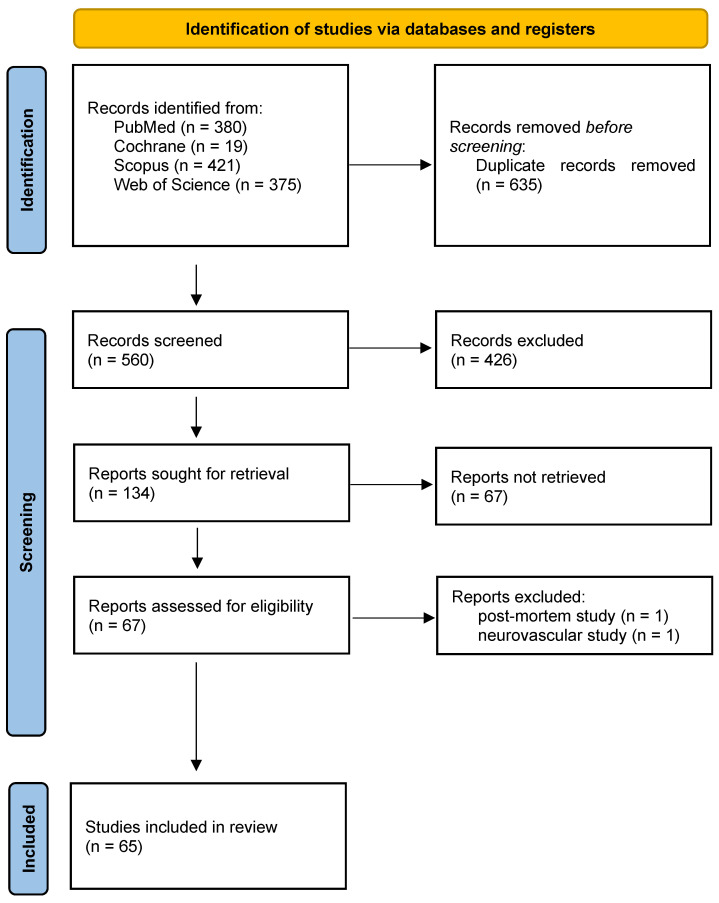
PRISMA flowchart.

**Figure 2:**
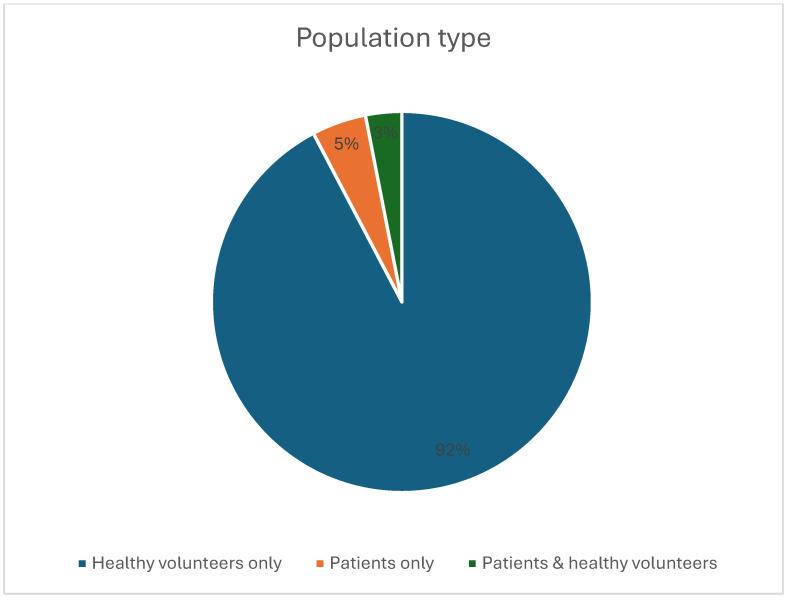
Pie chart showing the relative proportions of the type of cohort included in the studies.

A total of 65 studies meeting the inclusion criteria were identified (Figure 1), encompassing human in vivo 7-Tesla cardiovascular MRI investigations across multiple centers. The included studies demonstrated substantial heterogeneity in design, population, imaging objectives, and technical implementation. Most investigations involved small prospective cohorts, with a mean sample size of 13.0 ± 12.0 participants (median 10; range 3–72). Most studies enrolled healthy volunteers (Figure 2), while a smaller subset examined patient groups such as individuals with hypertrophic cardiomyopathy, carotid artery stenosis, or peripheral arterial occlusive disease [17,18,19,20]. All included studies are summarized in Supplementary File S1 (“study characteristics” sheet). Representative studies are cited within each application-specific subsection of the Results to illustrate key methodological and quantitative findings.

**Figure 3:**
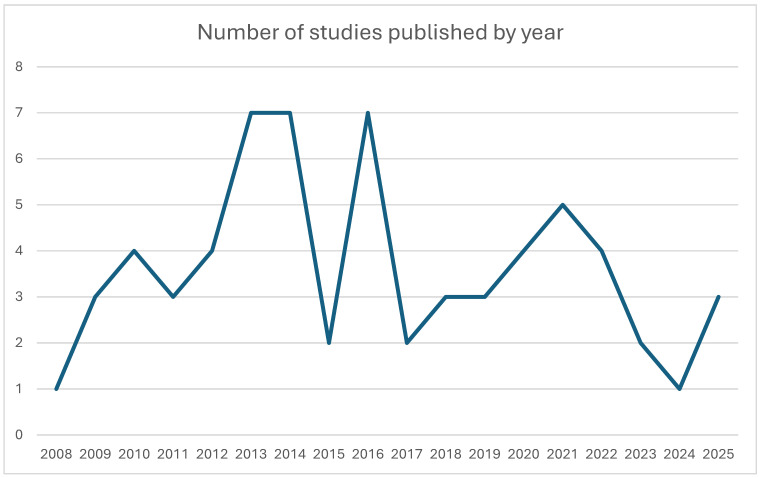
Line chart illustrating the number of clinical 7 T studies published each year from 2008 to 2025.

The annual number of published human 7 T CMR studies remained low throughout the study period, with fluctuating output and significant peaks in 2013–2014, 2016, and 2021, reflecting steady but slow growth in ultra-high-field cardiovascular research (Figure 3).

## 4. Study Characteristics

**Figure 4:**
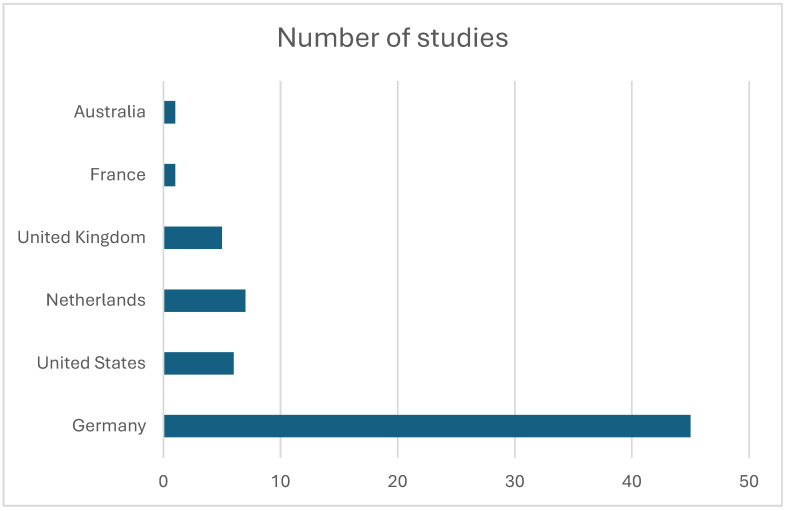
Histogram depicting the number of publications produced by each country.

Studies originated predominantly from specialized ultra-high-field research facilities in Europe and North America, including centers in the Netherlands, Germany, the United States, and the United Kingdom (Figure 4). Several research groups contributed multiple publications, reflecting active methodological development within dedicated 7 T laboratories (Table 1). The journals publishing these studies were primarily imaging and MR physics journals, with Magnetic Resonance in Medicine accounting for the largest proportion of publications (Table 2).

**Figure 5:**
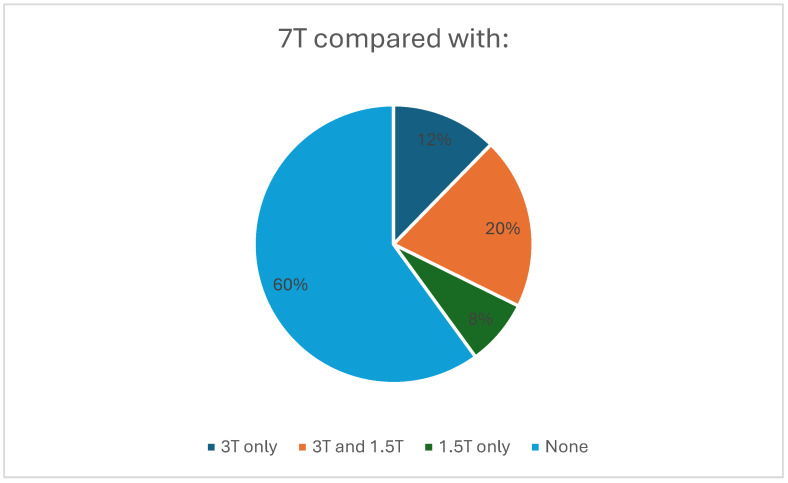
Pie chart showing the proportions of studies using no comparator, only 3 T, only 1.5 T, and both 1.5 T and 3 T as comparators.

The majority of included studies were non-comparison studies (60%) while 3 T was used as the principal comparator in most of the remaining studies (Figure 5).

## 5. Methodological Quality Appraisal

A structured qualitative methodological appraisal was performed for all included studies (Supplementary File S1: “methodological quality appraisal” sheet). Of the 65 included investigations, 39 (60%) were classified as exploratory feasibility studies, reflecting predominantly small sample sizes and an absence of comparator field strengths. Comparative feasibility designs were identified in 21 studies (32%), most commonly involving direct comparisons with 3 T systems. Only 5 studies (8%) met the criteria for quantitative validation, defined by the presence of comparator imaging, formal reproducibility metrics, and adequate sample size. Reproducibility analyses (e.g., ICC, coefficient of variation, Bland–Altman, or test–retest) were reported inconsistently across studies. In addition, safety reporting was heterogeneous and not consistently structured across studies, limiting the ability to perform formal safety synthesis or comparative tolerability assessment. Explicit blinded image analysis was infrequently described. Acquisition protocol transparency was generally high. Overall, these findings indicate that the current evidence base remains predominantly early-phase and methodological in nature.

## 6. Imaging Applications Evaluated

**Figure 6:**
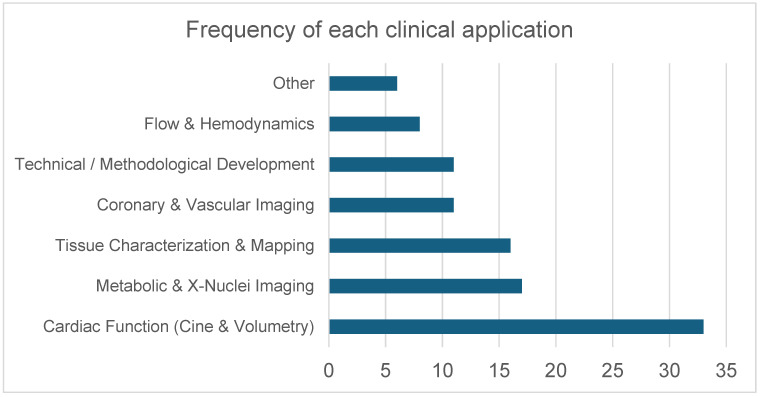
Distribution of cardiovascular MRI applications investigated at 7 T. Applications were grouped into six predefined domains: Cardiac Function (Cine & Volumetry) (including cine imaging, volumetric assessment, and cine-related deep learning/acceleration methods); Metabolic & X-Nuclei Imaging (^31^P spectroscopy, ^23^Na imaging, ^39^K imaging); Tissue Characterization & Mapping (T1 mapping, T2* mapping, fat–water imaging, perfusion imaging); Coronary & Vascular Imaging (coronary and peripheral MR angiography, carotid vessel wall imaging, small-vessel imaging, and aortic valve planimetry); Technical/Methodological Development (RF pulse design, B1 shimming, parallel transmission, coil development, acceleration techniques); and Flow & Hemodynamics (phase-contrast imaging, aortic 4D flow, and velocity mapping). Bars represent the number of studies addressing each application domain.

The included studies investigated a broad range of cardiovascular MRI applications at 7 T. This encompassed coronary MR angiography, cine imaging for ventricular function assessment, aortic valve planimetry, carotid vessel wall imaging, non-contrast perfusion imaging, thoracic aortic 4D flow, small-artery assessment in the hand, and multinuclear or spectroscopic acquisitions using ^31^P, ^23^Na, and ^39^K. Several studies also explored high-resolution fat–water imaging, T2* mapping, and metabolic evaluation using cardiac creatine kinase kinetics (Supplementary File S1: “study characteristics” sheet; Figure 6).

## 7. Main Findings Across Studies

### 7.1. Coronary and Vascular Imaging at 7 T

Coronary MR angiography at 7 T demonstrated improved quantitative image quality compared with 3 T in healthy volunteers. For right coronary artery (RCA) imaging, 7 T yielded a higher blood–epicardial fat contrast-to-noise ratio (87 ± 34 vs. 52 ± 13; *p* = 0.01), increased blood-pool signal-to-noise ratio (109 ± 47 vs. 67 ± 19; *p* = 0.02), and greater vessel sharpness (58% ± 9 vs. 50% ± 5; *p* = 0.04), while RCA diameter/length and navigator efficiency showed no field-strength–dependent differences. Left main coronary visualization was reported as less consistent than RCA, reflecting signal drop-off with surface-coil coverage [21].

In peripheral arterial occlusive disease, non–contrast-enhanced lower-leg MRA at 7 T was feasible and diagnostically accurate compared with 1.5 T contrast-enhanced MRA, with sensitivity of 93% and specificity of 98% for hemodynamically significant stenosis. Segment coverage at 7 T was lower (124/154 segments, 80.5%) due to incomplete iliac depiction in some patients related to fixed coil diameter [22]. For small-vessel applications, superficial palmar arch imaging at 7 T was feasible and achieved higher subjective vessel/vessel-wall visibility than 3 T MRI and micro-ultrasound [23]. In carotid vessel wall imaging, morphologic measurements were comparable between 7 T and 3 T (T1-weighted luminal area ICC 0.81; wall area ICC 0.84; T2-weighted luminal area ICC 0.97; wall area ICC 0.92), while 7 T produced significantly higher vessel-wall SNR and CNR (gain factors 1.3–3.6; *p* < 0.001 for T1-weighted and *p* < 0.05 for T2-weighted images), supporting improved plaque conspicuity potential [24]. Overall, coronary and vascular imaging studies suggest that 7 T provides measurable improvements in signal-to-noise ratio and vessel conspicuity, particularly in high-resolution structural applications. However, gains in quantitative vascular metrics were generally incremental rather than transformative, and technical factors such as coil coverage and field inhomogeneity continued to influence image uniformity and anatomic completeness.

### 7.2. Cardiac Function and Volumetry

Across multiple studies, cine cardiovascular magnetic resonance (CMR) at 7 T has been shown to be feasible and quantitatively comparable to clinical field strengths for the assessment of cardiac volumes, mass, and global function. Using FLASH and SSFP cine imaging, left ventricular (LV) volumes, ejection fraction, and mass did not differ significantly across 1.5 T, 3 T, and 7 T when identical sequence types were compared (all *p* > 0.05). However, systematic sequence-dependent differences were observed, with SSFP yielding larger end-diastolic and end-systolic volumes and lower LV mass than FLASH at all field strengths, including 7 T (*p* < 0.05). Quantitative LV measurements at 7 T demonstrated good agreement with 1.5 T, with intraclass correlation coefficients (ICC) ranging from 0.77 to 0.96 [25].

Functional cine imaging at 7 T was successfully performed in 80–100% of examinations across studies. Left ventricular diastolic filling assessment using velocity-encoded MRI showed strong agreement between 7 T and 1.5 T for transmitral stroke volume (ICC = 0.92) and early-to-atrial filling ratios (ICC = 0.77), with no significant inter-field differences. Cardiac chamber quantification using fast gradient-echo (FGRE) cine at 7 T demonstrated close agreement with SSFP at 1.5 T for LV volumes and ejection fraction, particularly when reduced slice thickness (4 mm vs. 7 mm) was employed [26].

Right ventricular (RV) cine imaging at 7 T was feasible, with diagnostic image quality in all subjects studied. RV end-diastolic volume, end-systolic volume, ejection fraction, and mass showed no significant differences compared with SSFP at 1.5 T (all *p* > 0.27), although a tendency toward mild RV volume overestimation at 7 T was reported with higher spatial resolution acquisitions [27].

In patients with hypertrophic cardiomyopathy, high-resolution 2D cine imaging at 7 T produced LV volumetric and functional parameters consistent with 3 T (all *p* > 0.09), while enabling detection of subtle myocardial crypts in 54% (7/13) of patients that were not visible at 3 T using standard clinical protocols [20].

Methodological advances enabled improved efficiency and robustness of cine imaging at 7 T. Simultaneous multi-slice (SMS) cine imaging achieved three-fold acceleration, with partial recovery of signal-to-noise ratio using regularized reconstruction (SNR 9.0 ± 4.5 vs. 10.1 ± 7.1 for single-slice cine), although the contrast-to-noise ratio remained reduced due to specific absorption rate-driven flip-angle limitations. Deep learning-based transfer learning approaches enabled accurate automated segmentation of 7 T cine data, improving Dice coefficients for LV and myocardium from 0.835/0.670 to 0.900/0.791, while requiring up to 90% fewer training images when using end-systolic and end-diastolic frames only [28]. Collectively, these findings indicate that 7 T cine imaging preserves quantitative agreement with established field strengths while offering enhanced structural detail. The primary advantage appears to lie in improved spatial resolution and myocardial delineation rather than in the alteration of global functional parameters.

### 7.3. Flow and Hemodynamics

Flow and hemodynamic assessment at 7 T were feasible across multiple studies, with successful acquisition of phase-contrast and 4D flow MRI data in the aorta and great vessels. High spatial resolution aortic 4D flow MRI at 7 T was enabled by advanced acceleration strategies and RF field optimization. Using kt-GRAPPA (R = 5), net acquisition time was reduced by a factor of 4.3 to approximately 10 min without significant differences in flow quantification compared with standard GRAPPA (R = 2), while higher spatial resolution (down to 1.2 × 1.2 × 1.2 mm^3^) allowed improved visualization of branching vessels. Optimization of B1^+^ shimming significantly influenced flow quantification (*p* < 0.05), underscoring the sensitivity of phase-based measurements to transmit field heterogeneity at 7 T [29].

The signal-to-noise ratio (SNR) for aortic 4D flow increased with field strength. In non-contrast-enhanced acquisitions, 7 T provided a 2.2-fold SNR increase over 3 T and a 3.7-fold increase over 1.5 T in the descending aorta. Contrast enhancement further increased SNR at all field strengths; however, relative gains were smaller at 7 T (1.4-fold) compared with 3 T (1.7-fold) and 1.5 T (1.8-fold). These SNR gains enabled higher spatial resolution and acceleration, but did not eliminate variability in quantitative flow metrics [30].

Comparative multi-field studies demonstrated that, although aortic 4D flow data could be acquired with sufficient diagnostic quality at 7 T, quantitative equivalence with lower field strengths was not consistently achieved. Non-diagnostic image quality was observed in 10 out of 144 aortic segments, 9 of which were acquired at 7 T. Significant differences across field strengths and sequence types were reported for forward flow (*p* < 0.001), wall shear stress (*p* < 0.05 to *p* < 0.001), and peak velocity (*p* < 0.001), despite moderate to strong correlations between measurements (r ≥ 0.5). Measurement variability across field strengths exceeded predefined equivalence limits based on interobserver variability [31].

Routine-style aortic flow measurements at 7 T were also demonstrated using commercially available hardware, with congruent stroke volume measurements obtained using both electrocardiographic and acoustic triggering. Non-diagnostic image quality affected a small proportion of ventricular segments (5.4% LV, 2.5% RV), and nominal flip angle significantly influenced right ventricular image quality. Reliable cardiac gating for combined cine and flow imaging at 7 T was achieved using vectorcardiographic trigger learning, yielding a sensitivity of 97.6% and specificity of 98.7% for R-wave detection, enabling quantitative assessment of aortic and pulmonary blood flow with mean image quality scores sufficient for analysis [32]. In summary, while 7 T enables high-resolution and accelerated flow imaging, quantitative hemodynamic equivalence across field strengths has not been consistently demonstrated. The incremental value of ultra-high-field flow imaging therefore appears to reside more in spatial refinement than in established parameter interchangeability.

### 7.4. Myocardial Tissue Characterization

Quantitative myocardial tissue characterization at 7 T was demonstrated using both relaxation mapping and compositional imaging, albeit with field-strength–specific technical constraints. Native myocardial T1 mapping was enabled by implementing adiabatic inversion for a ShMOLLI-based sequence (“ShMOLLI + IE”), allowing breath-hold acquisition within 22 heartbeats in healthy volunteers. Across the myocardium, inversion efficiency reached −0.79 to −0.83 (ideal −1), and native myocardial T1 was 1925 ± 48 ms [33].

Myocardial susceptibility-based characterization using T2*/R2* mapping was feasible at high spatial and temporal resolution at 7 T with dedicated shimming. Peak-to-peak B0 variation after volume-selective shimming was reduced to ~80 Hz (four-chamber/mid-ventricular views). Segmental analysis showed pronounced susceptibility-related limitations at longer echo times, with signal voids emerging for TE > 7 ms in anterior/inferior segments. Cine T2* mapping demonstrated significant cardiac cycle dependence, with T2* changes of up to ~27% between end-diastole and end-systole (*p* = 0.002). Complementary R2* analyses confirmed a linear increase with field strength and showed that artifact burden was greatest at 7 T versus 1.5 T (*p* = 0.010), with the inferior-lateral wall most affected, while the mid-septum remained relatively robust [34].

Compositional assessment via fat–water separation was also feasible for whole-heart 3D imaging under free-breathing conditions at 7 T using motion-compensated reconstruction. Respiratory motion correction reduced blurring and flow-related artifacts, and respiration-induced ΔB0 changes in motion-compensated maps were small (≈10 Hz on average) relative to the chemical-shift model used for separation [35]. Overall, myocardial tissue characterization at 7 T demonstrates enhanced sensitivity to relaxation and susceptibility effects, but also heightened vulnerability to field-dependent artifacts. The balance between increased contrast potential and technical instability remains application-specific.

### 7.5. Cardiac Metabolism and X-Nuclei Imaging

Ultra-high-field 7 T enabled multinuclear cardiac imaging and spectroscopy with measurable gains in signal and/or quantitative precision, supporting metabolic and ionic biomarkers beyond conventional ^1^H CMR. For ^31^P-MRS, matched acquisitions at 3 T and 7 T showed a 2.8× increase in PCr SNR at 7 T, accompanied by improved precision (CRLB reduced 2.4× for PCr concentration and 2.7× for PCr/ATP; PCr/ATP SD reduced ~2×). Myocardial ^31^P T_1_ values at 7 T were measured as follows: PCr 3.05 ± 0.41 s, γ-ATP 1.82 ± 0.09 s, α-ATP 1.39 ± 0.09 s, β-ATP 1.02 ± 0.17 s, and 2,3-DPG 3.05 ± 0.41 s [36].

Beyond static energetics, ^31^P saturation transfer approaches enabled in vivo quantification of myocardial creatine kinase (CK) kinetics at 7 T. Using 3D-localized BOAST, myocardial CK forward rate constants were measurable with kf = 0.35 ± 0.05 s^−1^; an alternative fast saturation transfer approach with 1D-ISIS/2D-CSI localization yielded kf = 0.29 ± 0.05 s^−1^, consistent with prior lower-field literature while highlighting dependence on transmit B1 inhomogeneity and localization strategy [37,38].

For ionic imaging, quantitative ^23^Na MRI demonstrated the importance of correction pipelines at 7 T: uncorrected myocardial tissue sodium concentration (TSC) was 54 ± 5 mM, which decreased by 48 ± 5% to 29 ± 3 mM after applying motion (cardiac/respiratory), B0/B1, and partial-volume corrections; partial-volume correction produced the largest reduction (34 ± 1%). Interleaved ^23^Na/^1^H acquisitions were feasible within clinically acceptable protocols, with modest penalties from combined coil setups (^23^Na B1^+^ efficiency −18.9%, ^23^Na SNR −15.4%) and improved ^1^H excitation homogeneity using customized pTx pulses (CV reduced from 0.37 with default shim to 0.30/0.23/0.15 for UPS/IPS/4kT, respectively), eliminating ^1^H signal dropouts [39]. First-in-human in vivo ^39^K MRI was also demonstrated at 7 T, achieving 14.5 mm isotropic nominal resolution in 30 min with mean cardiac SNR 9.6 ± 2.4 [40]. Taken together, multinuclear applications represent one of the clearest areas of added value for 7 T cardiovascular MRI, providing metabolic and ionic information that is difficult or impractical to obtain at lower field strengths.

## 8. Technical Parameters Reported

**Figure 7:**
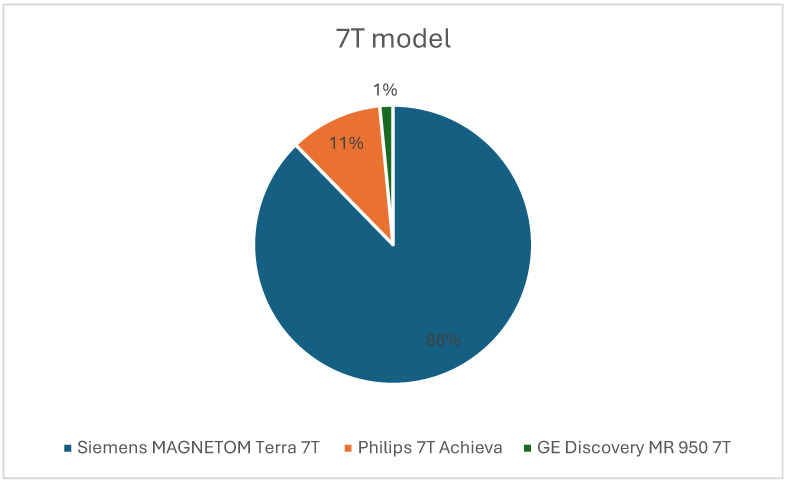
Pie chart showing the proportion of scanners used from each vendor.

Studies used a variety of scanner platforms, most commonly the Siemens MAGNETOM Terra 7 T system (Figure 7). Hardware configurations included single-channel transmit coils, multi-channel arrays, localized B1 shimming strategies, and parallel transmit (pTx) approaches. Investigators frequently reported B1^+^ inhomogeneity, SAR-related restrictions, and the need for tailored sequence modifications. Acquisition parameters were adjusted to accommodate ultra-high-field behavior, including inversion pulse design, shim optimization, respiratory and cardiac motion compensation, and multinuclear-specific localization methods.

## 9. Safety and Tolerability

Safety reporting was available in a portion of the included studies. When documented, 7 T cardiovascular MRI was generally well tolerated. Reported sensations included transient dizziness, a metallic taste, localized warmth or cold sensations, sleepiness, and mild paraesthesia. Occasional instances of vertigo during table movement, ECG waveform alterations due to magnetohydrodynamic effects, and localized skin redness were described. One participant discontinued scanning due to claustrophobia [21,25,27,41,42,43]. SAR values were consistently reported to remain within IEC regulatory limits, and studies utilizing pTx systems described adherence to established RF exposure constraints. No major adverse events were reported, and participant willingness to return for future 7 T examinations was high. However, the heterogeneity and inconsistency of safety documentation limit firm conclusions regarding tolerability across broader patient populations. Standardized prospective safety reporting frameworks will be essential in future multicenter studies to ensure robust evaluation of both acute and delayed adverse effects.

## 10. Discussion

This systematic review demonstrates that cardiovascular magnetic resonance imaging at 7 Tesla has progressed from early technical experimentation to a stage where repeatable, high-quality imaging is achievable across multiple cardiovascular applications. The included studies collectively show that the substantial signal-to-noise gains at 7 T can be leveraged for enhanced spatial resolution, improved tissue contrast, and more detailed visualization of cardiovascular structures that are challenging to resolve at 1.5 T or 3 T. These advances reflect sustained progress in radiofrequency coil design, pulse sequence development, and parallel transmission strategies, enabling applications spanning coronary and valvular imaging, functional assessment, vascular imaging, flow quantification, and multinuclear spectroscopy.

Across cine imaging studies, left- and right-ventricular volumes, mass, and ejection fraction at 7 T showed good agreement with measurements obtained at established clinical field strengths, indicating that ultra-high-field acquisition does not compromise fundamental cardiac quantification. Importantly, the principal advantage of 7 T cine imaging did not lie in altered global metrics, but rather in improved depiction of fine myocardial structures. Higher spatial resolution enabled clearer visualization of papillary muscles, trabeculations, and endocardial contours, and in selected disease cohorts revealed subtle myocardial features not visible at lower field strengths. These findings suggest that the incremental value of 7 T cine imaging is structural rather than volumetric and may be most relevant for detailed phenotypic characterization rather than routine functional assessment.

Coronary and vascular imaging results further support a selective advantage of 7 T for applications requiring high spatial detail. Coronary MR angiography at 7 T demonstrated higher blood–fat contrast, increased blood-pool signal-to-noise ratio, and improved vessel sharpness compared with 3 T, while measured vessel dimensions and navigator efficiency remained comparable. Similarly, carotid vessel wall imaging preserved morphologic agreement with 3 T while providing substantially higher signal-to-noise and contrast-to-noise ratios, a feature that may improve plaque conspicuity. Peripheral vascular studies showed that non-contrast-enhanced angiography at 7 T can achieve high diagnostic accuracy relative to contrast-enhanced imaging at lower field strengths, although incomplete anatomic coverage in some cohorts underscores the continued influence of coil geometry and penetration depth.

Flow and hemodynamic imaging at 7 T were feasible across multiple studies, with signal-to-noise gains enabling higher spatial resolution and accelerated 4D flow acquisitions. However, quantitative equivalence across field strengths was not consistently demonstrated for all flow-derived parameters, including peak velocity and wall shear stress. These findings indicate that, while 7 T facilitates high-resolution visualization of complex flow patterns, quantitative flow metrics remain sensitive to field-strength-specific effects such as transmit field inhomogeneity and sequence design. Further methodological standardization will therefore be required before 7 T flow measurements can be considered interchangeable with those obtained at conventional clinical field strengths.

Myocardial tissue characterization at 7 T was shown to be feasible using relaxation mapping, susceptibility-based techniques, and compositional imaging, but with notable field-specific constraints. Native T1 mapping and fat–water separation benefited from increased signal but required tailored pulse designs and careful B_0_/B_1_ optimization. Susceptibility-based techniques, including T2* mapping, demonstrated increased sensitivity at 7 T but also exhibited regional signal loss and cardiac-cycle-dependent variability, limiting uniform whole-heart assessment. These observations emphasize that ultra-high-field tissue characterization amplifies both contrast sensitivity and artifact susceptibility, necessitating application-specific optimization.

A distinct strength of 7 T cardiovascular MRI emerging from this review is its capability for multinuclear imaging and spectroscopy. Studies consistently demonstrated substantial improvements in signal-to-noise ratio and measurement precision for myocardial ^31^P spectroscopy, enabling in vivo assessment of high-energy phosphate metabolism and creatine kinase kinetics within practical scan times. The feasibility of cardiac ^23^Na imaging and first-in-human ^39^K imaging further illustrates the unique metabolic and ionic information accessible at ultra-high field, extending cardiovascular MRI beyond conventional morphological and functional assessment.

Despite these advances, technical limitations remain the principal barrier to broader clinical translation of cardiovascular MRI at 7 T. B_1_^+^ inhomogeneity and SAR-related constraints were consistently reported and directly influenced sequence selection, image uniformity, and acquisition efficiency. Balanced SSFP imaging remains challenging to implement routinely at ultra-high field, particularly for coronary applications, necessitating the use of alternative gradient-echo-based approaches in many studies [44]. In addition, cardiac gating is frequently affected by magnetohydrodynamic effects, although alternative triggering approaches demonstrated high reliability in several studies. Consequently, most successful examinations relied on tailored shimming strategies, parallel transmission, and application-specific workflows, limiting standardization and scalability in routine clinical environments.

Safety reporting across the included studies was reassuring, with no serious adverse events and only transient, mild sensations described. However, reporting practices were heterogeneous, and standardized documentation of tolerability and RF exposure will be essential as investigations move toward larger and more diverse patient cohorts.

Experience from coronary MR angiography at lower and intermediate field strengths further illustrates that increasing magnetic field strength does not uniformly translate into improved coronary image quality. Even at 3 T, coronary MRI is challenged by B_1_ field inhomogeneity, susceptibility effects, and SAR constraints that influence sequence selection and contrast-preparation strategies. While ultra-high-field systems offer substantial signal-to-noise gains, these gains may necessitate trade-offs in pulse design and magnetization preparation to remain within SAR limits, with potential consequences for vessel–myocardium contrast. Observations from 5 T coronary imaging exemplify this balance, demonstrating improved spatial resolution and depiction of calcified disease while also underscoring persistent limitations related to contrast preparation and signal uniformity. These considerations inform realistic expectations for coronary imaging at 7 T [45].

Against this backdrop, experience from intermediate ultra-high-field systems further informs the potential future role of 7 T cardiovascular MRI relative to emerging CT technologies such as photon-counting CT [46]. Studies at 5 T have demonstrated that non-contrast coronary MR angiography can leverage increased signal-to-noise ratio and spatial resolution to depict coronary anatomy and calcification without ionizing radiation or contrast agents, illustrating a trajectory in which higher field strengths may enable MRI to address selected coronary imaging tasks traditionally dominated by CT. Importantly, current high-field CMRA techniques remain limited in their ability to reliably characterize non-calcified atherosclerotic plaque, a domain in which CT-based techniques retain a clear advantage. Accordingly, ultra-high-field MRI is unlikely to replace CT for rapid population-level screening, but may instead assume a complementary, problem-solving role in complex or equivocal cases, radiation-sensitive populations, or scenarios requiring integrated assessment of coronary anatomy, myocardial structure, and tissue composition within a single examination [45].

Taken together, the current literature positions 7 T cardiovascular MRI as a powerful research and early translational platform rather than a replacement for established 1.5 T or 3 T systems. Its strengths lie in applications requiring ultra-high spatial resolution, detailed vessel wall or valvular assessment, and advanced metabolic characterization, whereas routine functional and flow quantification show more limited incremental benefit. Continued progress will depend on advances in RF management and parallel transmission, as well as the development of standardized acquisition protocols across vendors and centers to improve reproducibility and enable meaningful multicenter validation. Protocol harmonization and disease-focused studies will be essential to determine whether the additional information provided by 7 T translates into improved diagnostic confidence or clinical decision-making.

**Figure 8:**
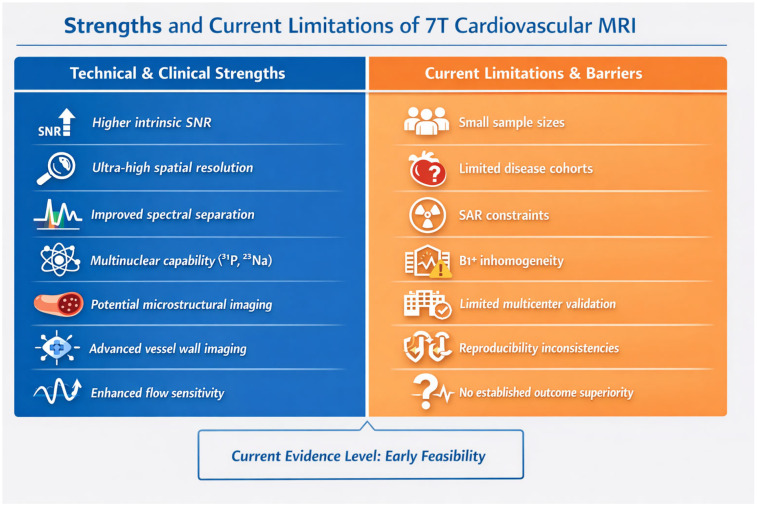
Strengths and current limitations of 7 T cardiovascular MRI.

Importantly, the overall strength of evidence should be interpreted as early feasibility level. Although several studies demonstrate technical reproducibility and cross-field agreement, the predominance of small single-center investigations and healthy volunteer populations limits generalizability. Only a minority of studies fulfilled criteria for quantitative validation. Therefore, current evidence supports technical feasibility rather than established clinical superiority. Consequently, the level of evidence should be regarded as exploratory, and conclusions regarding clinical benefit should be considered hypothesis-generating rather than definitive. The principal strengths and current limitations of 7 T cardiovascular MRI identified in this review are summarized in Figure 8.

This review has several limitations. First, the available evidence is predominantly derived from small, single-center feasibility studies with limited sample sizes and a predominance of healthy volunteers. Comparative studies with established clinical field strengths were inconsistent, and formal diagnostic accuracy or outcome-based endpoints were rarely evaluated. Considerable heterogeneity in scanner platforms, coil configurations, pulse sequence implementations, and parallel transmission strategies further limits cross-study comparability and generalizability. In addition, reproducibility metrics and standardized quantitative benchmarks were variably reported, and a formal risk-of-bias tool was not applied due to the technical and exploratory nature of most included investigations. Second, limitations related to the review process should be acknowledged. The protocol was not prospectively registered, and only English-language publications were included, introducing potential language bias. Conference abstracts and grey literature were not systematically searched, which may increase the risk of publication bias. Finally, substantial methodological heterogeneity precluded meta-analysis; therefore, results were synthesized narratively.

## 11. Conclusions

Cardiovascular MRI at 7 Tesla is technically feasible and has demonstrated promising reproducibility in selected applications. However, the current evidence base remains predominantly early-phase and exploratory, with small sample sizes and a predominance of healthy volunteer cohorts. While quantitative agreement with lower field strengths has been shown in several domains, clinical superiority and outcome impact have not yet been established. Larger, disease-focused, multicenter validation studies are required before routine clinical adoption can be justified.

## Figures and Tables

**Table 1:** List of most frequent first authors and the labs to which they were affiliated during the study.

First Author	Count	Lab
Sebastian Schmitter	4	Center for Magnetic Resonance Research, Department of Radiology, University of Minnesota, USA
Christoph Stefan Aigner	3	Physikalisch-Technische Bundesanstalt (PTB), Braunschweig and Berlin, Germany
Andreas Graessl	3	Berlin Ultrahigh Field Facility, Max-Delbrück-Center for Molecular Medicine, Germany
William T. Clarke	3	Wellcome Centre for Integrative Neuroimaging, FMRIB, Nuffield Department of Clinical Neurosciences, University of Oxford, UK
Till Huelnhagen	2	Berlin Ultrahigh Field Facility, Max Delbrück Center for Molecular Medicine, Germany
Florian von Knobelsdorff-Brenkenhoff	2	Berlin Ultrahigh Field Facility, Max-Delbrück-Center for Molecular Medicine, Germany
Christopher T. Rodgers	2	Oxford Centre for Clinical Magnetic Resonance Research (OCMR), University of Oxford, UK
Anja Fischer	2	University Hospital Essen, Germany
Tobias Frauenrath	2	Berlin Ultrahigh Field Facility, Max-Delbrück-Center for Molecular Medicine, Germany
Saskia G. C. van Elderens	2	Leiden University Medical Centre, Netherlands

**Table 2:** List of journals and their respective number of published papers relative to clinical 7 T cardiovascular imaging.

Journal	Count
Magnetic Resonance in Medicine	33
PLOS ONE	5
Investigative Radiology	4
Journal of Magnetic Resonance Imaging	3
Journal of Cardiovascular Magnetic Resonance	3
NMR in Biomedicine	2
World Journal of Radiology	2
European Journal of Radiology	2
European Radiology	2
Radiology	1
Frontiers in Cardiovascular Medicine	1
Journal of Magnetic Resonance	1
Magnetic Resonance Imaging	1
Current Cardiology Reviews	1
Journal of Visualized Medicine	1
Tomography	1
Scientific Reports	1

## Data Availability

The database used for this study is available through Figshare at https://doi.org/10.6084/m9.figshare.30877619.

## Supplementary Materials

The following supporting information can be downloaded at https://www.mdpi.com/article/10.3390/diagnostics16060937/s1: Supplementary File S1: Study characteristics and methodological quality appraisal sheets, Supplementary File S2: Study Protocol, Supplementary File S3: PRISMA 2020 Checklist.
