# Supplementary material for: Human In Vivo Cardiac Magnetic Resonance Imaging at 7 T: Feasibility, Applications, and Current Limitations—A Systematic Review
Source: Diagnostics (Basel). 2026 Mar 22;16(6):937. doi: 10.3390/diagnostics16060937 (PMC13025201; doi:10.3390/diagnostics16060937)
Supplement: Supplementary file 1 [file diagnostics-16-00937-s001.zip › supplemetary File S2.pdf]

Protocol for Systematic Review

Human In-Vivo Cardiovascular Magnetic Resonance Imaging at 7 Tesla: Feasibility, Applications and Current Limitations

1. Background and Rationale

Ultra-high-field magnetic resonance imaging at 7 Tesla (7T) provides substantially increased intrinsic signal-to-noise ratio (SNR), which may enable improved spatial resolution, enhanced tissue contrast, and advanced multinuclear and spectroscopic applications in cardiovascular magnetic resonance (CMR). However, translation into routine clinical cardiovascular imaging remains limited by technical challenges including radiofrequency (RF) inhomogeneity, specific absorption rate (SAR) constraints, sequence instability, and gating interference.

Although individual human in-vivo studies have demonstrated feasibility across multiple cardiovascular applications, no comprehensive synthesis of the current clinical experience has systematically evaluated:

- Feasibility and safety
- Quantitative reproducibility
- Comparator performance vs 1.5T/3T
- Application-specific strengths and limitations
- Methodological rigor of the existing literature

This systematic review aims to synthesize and critically appraise the current human in-vivo evidence base.

2. Objectives

Primary Objective

To evaluate the feasibility and technical performance of 7T cardiovascular MRI in human in-vivo studies.

Secondary Objectives

1. To characterize application-specific performance (e.g., cine imaging, coronary MRA, flow imaging, tissue characterization, multinuclear imaging).
2. To evaluate quantitative agreement with conventional field strengths (1.5T and 3T), where comparators exist.
3. To assess methodological rigor and reporting quality of included studies.
4. To identify recurrent technical limitations and barriers to clinical translation.
5. To summarize safety and tolerability findings.

3. Review Framework

The review follows a **PIRD diagnostic framework**:

- **Population (P):** Human participants undergoing cardiovascular MRI.
- **Index Test (I):** 7-Tesla cardiovascular MRI.
- **Reference Standard (R):** 1.5T or 3T MRI when available.
- **Domain (D):** Feasibility, image quality, quantitative performance, technical implementation, safety.

A PICO framework was not used due to the exploratory and heterogeneous nature of early-phase feasibility studies.

4. Eligibility Criteria

Inclusion Criteria

Studies were eligible if they:

1. Included human in-vivo cardiovascular MRI performed at 7 Tesla.
2. Reported at least one of:
    - Anatomical imaging outcomes
    - Functional assessment
    - Flow/hemodynamic measurements
    - Quantitative tissue characterization
    - Image quality metrics
    - Clinical feasibility or safety
3. Were original peer-reviewed research articles.
4. Were published in English.

Exclusion Criteria

- Animal, ex-vivo, phantom-only studies
- Pure engineering or simulation studies without human imaging
- Intracranial neurovascular imaging studies
- Organ-specific non-cardiac vascular imaging (e.g., renal-only studies)
- Conference abstracts without full manuscripts

5. Information Sources

The following databases were searched from inception to January 2025:

- PubMed
- Cochrane Library
- Web of Science
- Scopus

Reference lists of eligible studies were screened manually.

6. Search Strategy

A structured search string was developed using combinations of:

- "magnetic resonance imaging" OR "MRI"
- "7 Tesla" OR "7T" OR "ultra high field"
- "cardiac" OR "cardiovascular" OR "coronary" OR "myocardium"

Search strings were adapted for each database.

7. Study Selection

Screening Process

- Titles and abstracts were independently screened by two reviewers.
- Full-text screening was performed independently by the same reviewers.
- Disagreements were resolved by structured discussion.
- If consensus could not be reached, a senior reviewer adjudicated.

A PRISMA 2020 flow diagram documents selection.

8. Data Extraction

Data extraction was performed independently by two reviewers using a predefined standardized extraction form.

Extracted variables included:

- Study design
- Sample size
- Population characteristics
- Comparator presence
- Imaging application
- Technical setup (scanner, coil, pTx use)
- Feasibility success rate

- Quantitative measurements
- Reproducibility metrics (ICC, CV, test–retest)
- Safety reporting
- Reported limitations

Discrepancies were resolved by consensus review of the full text.

Incomplete reporting was not imputed; missing data were recorded as “not reported.”

9. Definition of Feasibility and Endpoints

Given heterogeneity across studies, feasibility was operationally defined as:

- Successful acquisition of interpretable diagnostic images
- Completion of intended protocol in ≥80% of participants
- Quantitative measurement derivable from acquired data

Image quality endpoints were accepted as defined by individual studies, including:

- SNR / CNR
- Vessel sharpness
- Visual grading scales
- Diagnostic segment coverage

Reproducibility endpoints included ICC, coefficient of variation, and agreement metrics when reported.

10. Methodological Quality Assessment

Given the technical translational nature of ultra-high-field MRI research, traditional diagnostic risk-of-bias tools (e.g., QUADAS-2) were deemed methodologically inappropriate; therefore, a domain-based structured quality appraisal tailored to imaging feasibility research was prespecified assessing:

1. Sample size adequacy
2. Inclusion of comparator field strength
3. Reporting of reproducibility metrics
4. Reporting of safety outcomes
5. Transparency of acquisition protocol
6. Blinding of image analysis (when applicable)

Each study was categorized as:

- **Exploratory Feasibility**

- **Comparative Feasibility**
- **Quantitative Validation Study**

This framework was used to contextualize strength of evidence.

11. Data Synthesis

Due to substantial heterogeneity in:

- Study design
- Imaging protocol
- Field-strength comparators
- Outcome metrics

Meta-analysis was not planned.

Data were synthesized narratively and grouped by application domain:

- Coronary and vascular imaging
- Cine imaging
- Flow imaging
- Tissue characterization
- Multinuclear/metabolic imaging

Evidence strength was interpreted as **early-phase feasibility** unless comparative validation was demonstrated.

12. Protocol Deviations

No substantive deviations from this protocol occurred.

Minor clarifications to eligibility wording were made during full-text review to improve operational consistency but did not alter scope, outcomes, or inclusion criteria.

13. Registration

This protocol was developed a priori before database searches commenced.
It was not prospectively registered in PROSPERO.

The protocol is provided publicly as Supplementary Material to ensure transparency and enable assessment of methodological consistency.

14. Amendments

Any future protocol amendments will be documented in updated versions of the supplementary file.
